# Clot or not? An unusual case of false positive CTPA and an approach to diagnosis

**DOI:** 10.1259/bjrcr.20160021

**Published:** 2016-07-25

**Authors:** Cheryl Main, Ausami Abbas, James S Shambrook, Charles Peebles, Stephen Harden

**Affiliations:** ^1^Department of Radiology, University Hospital Southampton, Southampton, UK; ^2^Department of Cardiothoracic Radiology, University Hospital Southampton, Southampton, UK

## Abstract

The case involves a 69-year-old female with severe, longstanding bronchiectasis secondary to childhood pertussis infection. She presented to the hospital and was thought clinically to have a pulmonary embolus. A CT pulmonary angiogram was performed, which was technically satisfactory. This revealed multiple, bilateral filling defects that were fairly convincing for pulmonary emboli. Further review of the CT scan not only revealed the extent of her bronchiectasis but also a number of enlarged bronchial arteries supplying the diseased lung. The pulmonary arterial filling defects arose suspiciously close to the bronchial arteries and the possibility of bronchial to pulmonary artery anastomoses was considered. Could the admixture of highly contrast-opacified pulmonary arterial blood with partially opacified systemic arterial blood cause the apparent filling defects? After further consideration, a second electrocardiography-gated CT angiogram was performed—this time in the systemic arterial phase but planned with two regions of interest sited over the main pulmonary artery and the aorta with the aim of triggering the scan with maximum contrast in the bronchial arteries, and as much contrast washout as possible in the pulmonary arteries. This study revealed a reversal of the CT pulmonary angiogram appearances with contrast now seen in the bronchial arteries and opacifying the sites of the previous filling defects in the pulmonary arteries. Thus, the filling defects were actually false positives caused by an admixture of highly opacified and part-opacified blood via bronchial artery anastomoses. In the context of a false-positive finding of pulmonary embolus on a background of severe bronchiectasis, unnecessary anticoagulation could have increased the risk of complications such as haemoptysis. This case report illustrates the importance of knowledge of potential false-positive findings in CT pulmonary angiography and describes a novel approach based on cardiac CT techniques to prove this.

## Clinical presentation

A female patient in her sixties had chronic bronchiectasis following pertussis infection during childhood. She suffered frequent chest infections, occasionally requiring hospitalization. She presented to the emergency department with an episode of acute shortness of breath on minimal exertion and new left-sided pleuritic chest pain. Following admission under the respiratory team, she was found to be tachycardic, tachypnoeic and hypoxic. Arterial blood gas taken with no supplemental oxygen revealed a pH of 7.4, pAO_2_ of 8.78 and pACO_2_ of 5.48. Laboratory blood tests showed a raised white cell count of 14.9 k μl^−1^ and a C-reactive protein level of 133 mg l^−1^.

The patient was commenced on antibiotic treatment for presumed lower respiratory tract infection but on this occasion, her symptoms were different from her usual presentation, and pulmonary embolism was considered as the main differential.

## Imaging findings

**Figure 1.**
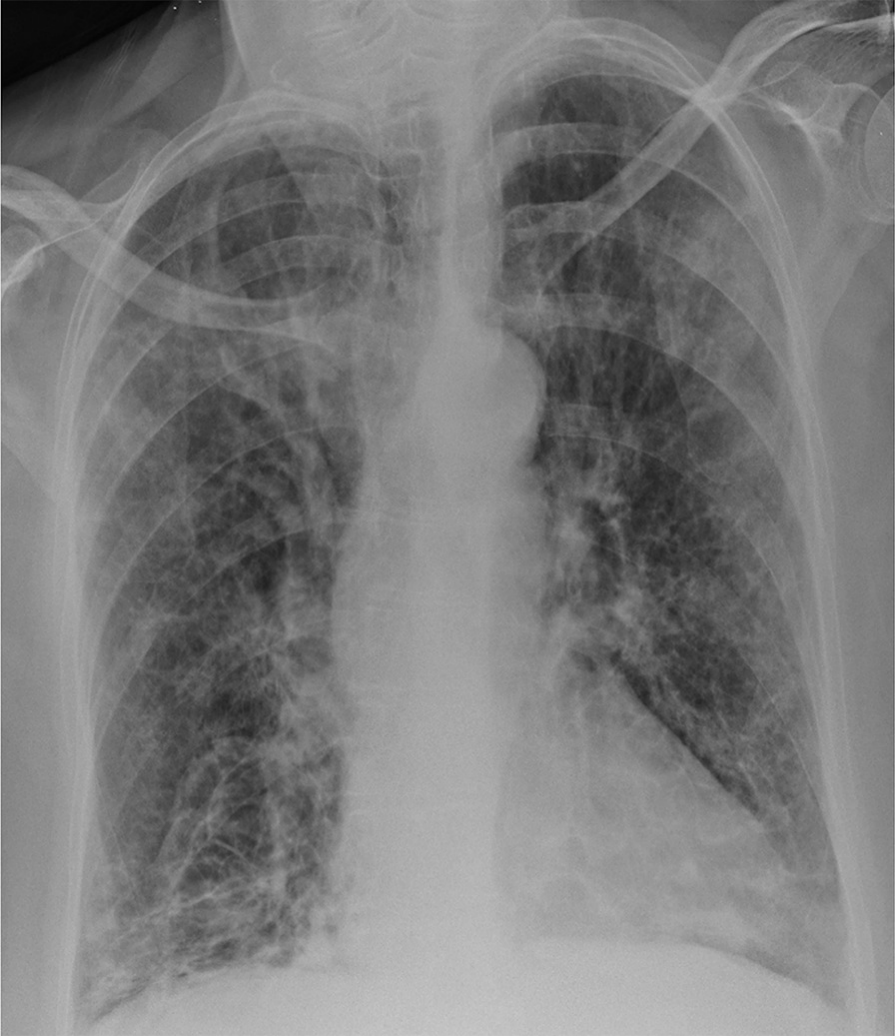
Posteroanterior chest radiograph performed acutely demonstrating hyperexpansion, ring and tram line shadows, coarse lung markings and architectural distortion. No new changes were seen compared with previous radiographs.

The initial chest X-ray (Figure 1) showed ring and tram line shadows, which was consistent with bronchiectasis but no significant changes were seen compared with previous radiographs.

**Figure 2.**
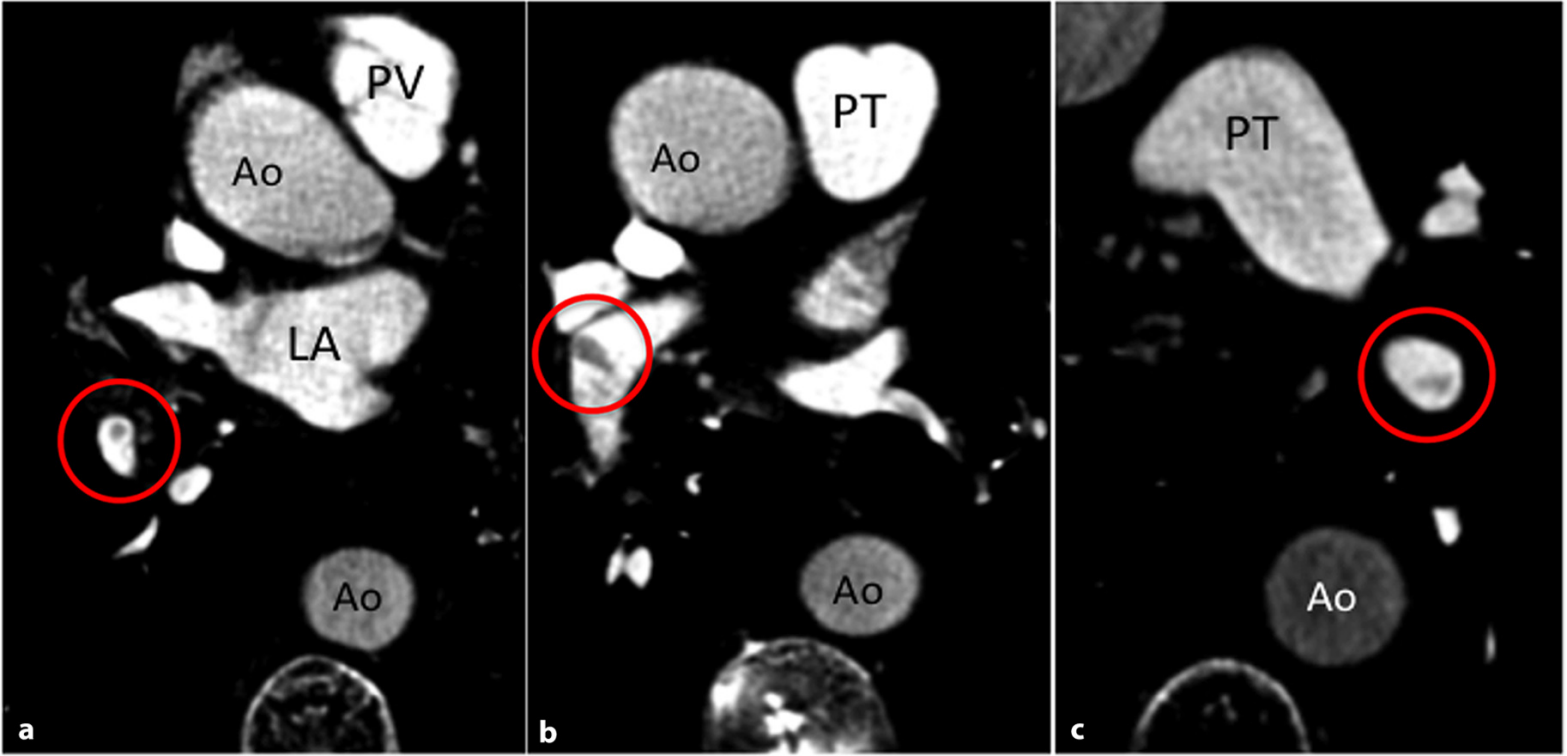
(a–c) CT pulmonary angiogram. Selected axial slices with optimization of windowing for pulmonary arteries. Filling defects are visible within multiple pulmonary arteries (red circles). Ao, aorta; LA, left atrium; PT, pulmonary trunk; PV, pulmonary vein.

**Figure 3.**
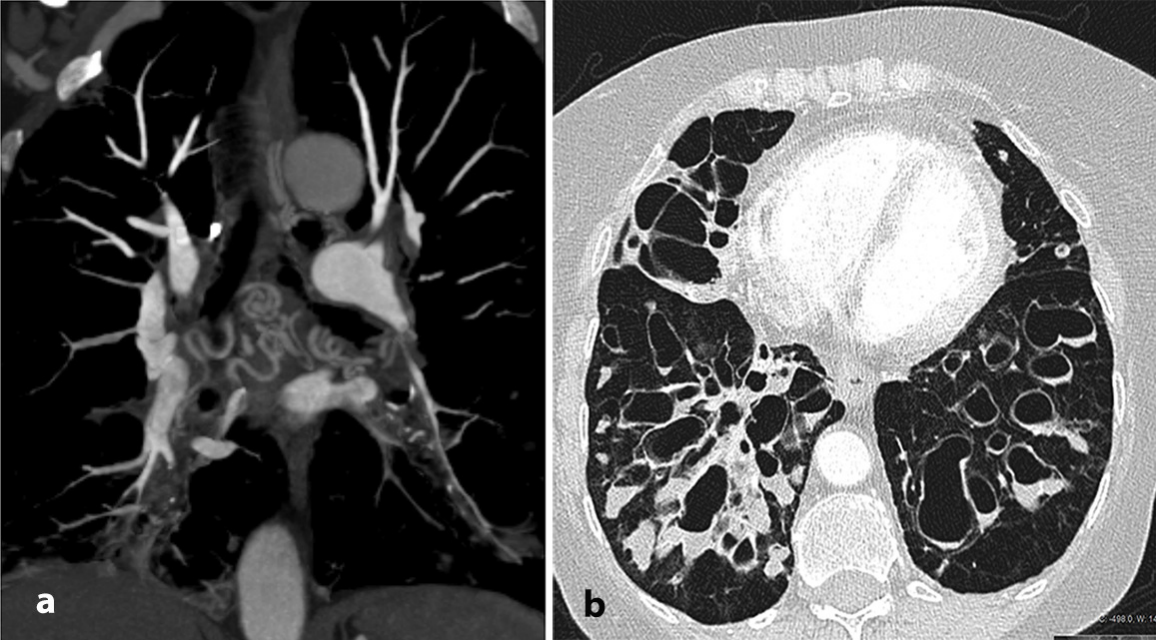
(a) Coronal maximum intensity projection reformat of the mediastinum from the intitial CT pulmonary angiogram shows multiple collateral bronchial arteries supplying the diseased lung. (b) Axial slice of the same CT scan with lung windowing shows severe cystic bronchiectasis, bronchial wall thickening and bronchocoeles/intrabronchial fluid levels.

A CT pulmonary angiogram (CTPA) was performed, which demonstrated multiple pulmonary artery filling defects (Figure 2a–c). Review of lung windows of the same study revealed numerous bronchial artery collaterals (Figure 3a) supplying the diseased lung and severe cystic bronchiectasis (Figure 3b).

On initial review, the imaging features suggested multiple pulmonary emboli but the filling defects showed a close anatomical relationship with bronchial artery collaterals. This raises the possibility that the filling defects could actually be a result of admixture of unopacified blood from bronchial artery collaterals anastomising with, and flowing into, the contrast filled pulmonary arteries. To prevent unnecessary anticoagulation in a patient at risk of haemorrhage from bronchiectasis, a tailored low-dose study was performed using a cardiac CT protocol with a controlled intravenous iodinated contrast bolus injected at 5.5 ml s^−1^ followed immediately by a saline bolus to provide relative negative contrast in the pulmonary arteries. This was acquired in the systemic arterial phase, and electrocardiography gating was used to minimize cardiac motion artefact and optimize resolution of the bronchial artery collaterals.

**Figure 4.**
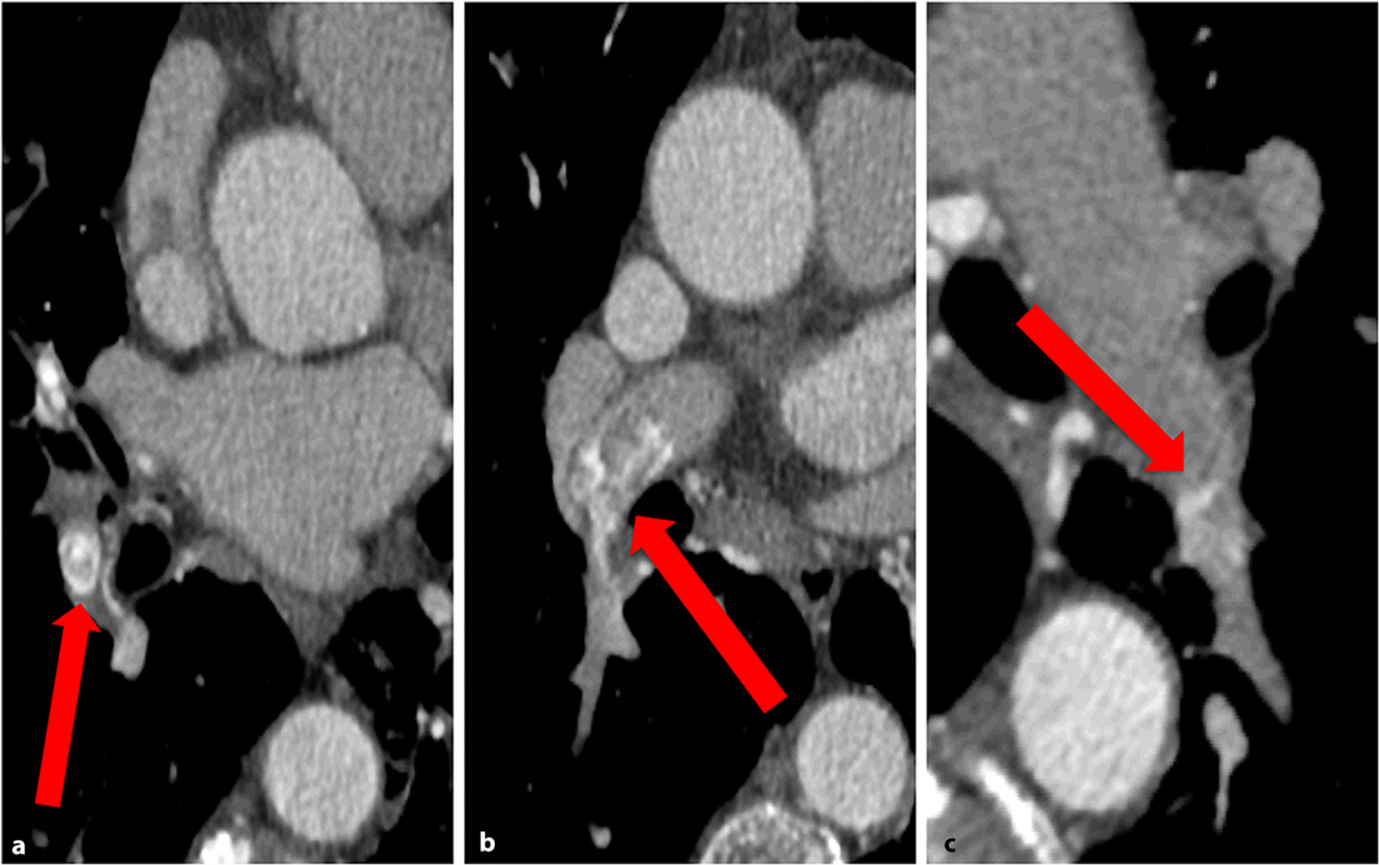
(a–c) Axial slices from the CT aortic angiogram showing reversal of the initial findings with systemic arterial contrast forming a positive filling defect against the relative negative contrast in the pulmonary arteries (red arrows).

**Figure 5.**
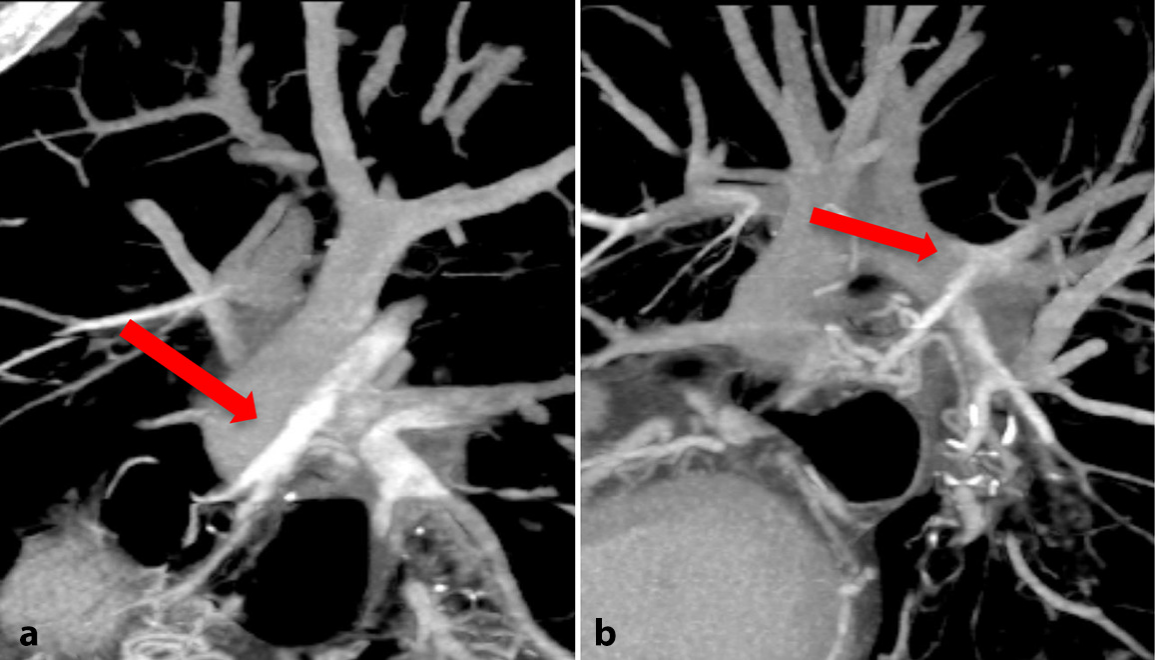
(a) Curved reformat images from CT aortic angiogram showing heavily opacified bronchial arteries (red arrow) anastomosing with pulmonary arteries. (b) A blush of contrast is seen spilling into the relative negative contrast in the pulmonary arterial circulation (red arrow).

This study revealed a reversal of the CTPA appearances, with contrast now seen in the bronchial arteries and opacifying the sites of the previous filling defects in the pulmonary arteries (Figure 4). These findings verified with curved reformats using maximum intensity projections (Figure 5) confirmed that the filling defects were actually false positives from an admixture of highly opacified and part-opacified blood from the bronchial artery anastomoses flowing into the pulmonary arterial circulation.

## Treatment

On the basis of these findings, the treatment dose of anticoagulant was terminated, mitigating the significant risk of haemorrhage in a patient with severe bronchiectasis. She recovered following a course of antibiotics and was discharged home.

## Discussion

CTPA is well established as the imaging technique of choice for the assessment of possible thromboembolic disease.^1^ CTPA provides diagnostic information for other thoracic structures in addition to the appearances of the pulmonary arterial tree. It is not uncommon that alternative diagnoses are found to account for symptoms; indeed, this was reported in 28% of cases in a recent study.^2^ CTPA is accurate in identifying pulmonary emboli with a reported sensitivity of 83% and specificity of 96%.^3^ False positives are therefore uncommon but are well recognized.^4–6^ In this case, the formation of anastomoses is unusual but provides an alternative explanation for the filling defects seen on the CTPA, a hypothesis tested and confirmed by an individualized electrocardiography-gated thoracic aortic-type CT protocol. This case highlights the need to scrutinize all apparent filling defects of the pulmonary artery tree seen on CTPA to exclude false-positive diagnoses.

Causes of false-positive results on CTPA:^4–7^

1. Pathological:
  - Tumour embolus
  - Angiosarcoma
  - Arteritis
  - Proximal interruption of the pulmonary artery
  - Post ligation of pulmonary artery/lobectomy
2. Iatrogenic:
  - Cavopulmonary anastomoses
3. Technical:
  - Admixture/flow
  - Beam hardening
  - Respiratory motion
  - Image noise
  - Partial volume artefact
4. Interpretational:
  - Window settings
  - Misinterpretation of veins
  - Crossing vessels
  - Adjacent lymphoid tissue
  - Adjacent mucous plug
  - Vascular bifurcation
  - Perivascular oedema

## Learning points

1. CTPA is a useful study for diagnosing pulmonary embolism and can demonstrate alternative diagnoses where the pulmonary arteries are normal.
2. Careful analysis of pulmonary arterial filling defects should be performed to exclude false positives.
3. An unusual cause of false positives on CTPA is anastomosis with bronchial arteries.
4. Tailored low-dose systemic arterial phase CT studies can be considered to exclude such anastomoses.

## Consent

The patient has signed a specific consent form indicating the nature of the case report and the purpose for which it is intended.
